# Logometro^®^: The psychometric properties of a norm-referenced digital battery for language assessment of Greek-speaking 4–7 years old children

**DOI:** 10.3389/fpsyg.2022.900600

**Published:** 2022-07-26

**Authors:** Faye Antoniou, Asimina M. Ralli, Angeliki Mouzaki, Vassiliki Diamanti, Sofia Papaioannou

**Affiliations:** ^1^Department of Educational Studies, National and Kapodistrian University of Athens, Athens, Greece; ^2^Department of Psychology, National and Kapodistrian University of Athens, Athens, Greece; ^3^Department of Primary Education, University of Crete, Rethymno, Greece; ^4^Department of Special Needs Education, University of Oslo, Oslo, Norway; ^5^School of Humanities, Hellenic Open University, Patra, Greece

**Keywords:** screening, digital assessment tool, developmental language disorder, specific learning disabilities, oral language, emergent literacy skills

## Abstract

In educational and clinical settings, few norm-referenced tests have been utilized until now usually focusing on a single or a few language subcomponents, along with very few language rating scales for parents and educators. The need for a comprehensive language assessment tool for preschool and early school years children which could form the basis for valid and reliable screening and diagnostic decisions, led to the development of a new norm-referenced digital tool called Logometro®. The aim of the present study is to describe Logometro® as well as its psychometric characteristics. Logometro® evaluates an array of oral language skills across the different language domains such as phonological awareness, listening comprehension, vocabulary knowledge (receptive and expressive), narrative speech, morphological awareness, pragmatics, as well emergent literacy skills (letter sound knowledge and invented writing) in Greek-speaking 4–7 years old children. More specifically, Logometro® has been designed in order to: (a) map individual language development paths as well as difficulties, (b) provide a descriptive profile of children’s oral language and emergent literacy skills, and (c) assist in the identification of children who are at risk for Developmental Language Disorder (DLD) or Specific Learning Disabilities (SLD). The sample consisted of 926 children aged from 4 to 7 years, which were recruited from diverse geographical provinces and represented a variety of socioeconomic backgrounds in Greece. Eight hundred participants were typically developing children (*N*_boys_ = 384 and *N*_girls_ = 416), 126 children (*N*_SLI_ = 44 and *N*_SLD_ = 82) represented children with Special Educational Needs, and 126 children were typically developing peers matched for gender and age with the clinical groups. The administration lasted 90 min, depending on the participant’s age and competence. Validity (construct, criterion, convergent, discriminant, and predictive) as well as internal consistency and test–retest reliability were assessed. Results indicated that Logometro® is characterized by good psychometric properties and can constitute a norm-referenced battery of oral language and emergent literacy skills. It could be used to inform the professionals as well as the researchers about a child’s language strengths and weaknesses and form the basis on which they can design an appropriate individualized intervention if needed.

## Introduction

Oral language and literacy skills are the principal tools for understanding and communicating with the world. They set the foundations for children’s academic achievement (Dickinson and Porche, 2011) and social development (Lindsay and Dockrell, 2012; Snow and Powell, 2012). Nevertheless, language delays and literacy difficulties comprise the most common problems during the preschool years and first grades of primary school (Tomblin et al., 1997). Early identification is therefore inextricably linked to early intervention, in order to address and support children with language difficulties at this early developmental stage (Fletcher et al., 1994; Stanovich and Siegel, 1994; Francis et al., 1996; Morris et al., 1998).

Language difficulties can be related to deficits in various domains such as non-verbal cognitive abilities (Buschmann et al., 2008), motor skills (Finlay and McPhillips, 2013), social competence (Mok et al., 2014), as well reading and writing skills (Catts et al., 2002). Such difficulties raise the first concerns for the children who may get a diagnosis for neurodevelopmental disorders (Kozlowski et al., 2011; Jansen et al., 2021) such as Developmental Language Disorder (DLD) and Specific Learning Disabilities (SLD), later in Primary school.

Developmental Language Disorder defines a condition in which difficulties with language acquisition (understanding and/or spoken) persist until adulthood (Leonard, 2014). DLD is not linked to intellectual impairment, hearing disability, or autism spectrum disorder (Leonard and Deevy, 2020). The most outstanding indicators of DLD, during the preschool years and early school years, are deficits in phonological awareness (Snowling et al., 2019) and morphosyntactic awareness, as well as vocabulary delays (Leonard and Deevy, 2020).

Specific Learning Disabilities are identified when a child does not meet age- or grade-level standards in one or more of the following areas: oral expression, listening comprehension, written expression, basic reading skills, reading fluency, reading comprehension and mathematics (IDEA, 2004). Children who are diagnosed with SLD in Primary school, often had oral language difficulties during the preschool years, regarding semantic, grammatical, and phonological awareness skills (Muter et al., 2004). Such deficits are related to a high risk for a diagnosis of reading disabilities (Bradley and Bryant, 1983).

Even though DLD and SLD are separable conditions varying in severity, as defined by DSM-V, they share common underlying mechanisms and they can co-exist (Bishop et al., 2009; Snowling et al., 2019). Consequently, identifying language difficulties in the early years helps professionals working with preschoolers to describe children’s oral language profile requiring a clinical referral or reach a diagnostic decision and respond to their needs (Jansen et al., 2021). Especially in early childhood when growth accelerates at a high speed, differentiating between typical and atypical language development can be challenging (Egger and Angold, 2006). Thus, the heterogeneity at these ages exemplifies the diagnostic complication with developmental difficulties. Therefore, there is a need for the development of valid and reliable tools for language assessment, in order the professionals to reliably assess children’s oral language and literacy skills and reach safe conclusions.

Language assessment tools usually focus on one or more language subcomponents, such as phonology, vocabulary knowledge, narration, morphology, and pragmatics (e.g., CELF-5, Wiig et al., 2013; Preschool Language Scale, Zimmerman et al., 1979). These language sub components have a discrete effect on overall language competence and -when combined- predict cognitive or academic difficulties (Mouzaki et al., 2020). However, there are not many standardized psychometric tools for Greek-speaking children, and not only, that utilize all language subcomponents in order to provide a holistically, psychometrically sound and clinically accurate profile. The lack of a valid and reliable language measure for preschool and early school years children led to the development and standardization of Logometro.

Greek is a language with relatively consistent orthography (Protopapas and Vlahou, 2009), as well as rich inflectional and derivational morphology (Ralli, 2003). Nouns and adjectives in Greek are obligatorily inflected for gender, number, and case *via* fusional suffixation. Verb forms also include a stem and an obligatory inflectional ending, both of which may be simple or complex. Verbs are inflected for voice, aspect, tense, number, and person (Ralli, 2003; see Klairis and Babiniotis, 2004; Holton et al., 2012, for comprehensive descriptions). As previously described (see Diamanti et al., 2017) most Greek-speaking children in Preschool are able to improve their phonological awareness skills if they get practice and they approach ceiling performance in the production of verb past tense and noun gender, number, and case (Mastropavlou, 2006). Nevertheless, persistent difficulties with verb aspectual formation and noun gender are observed in certain word classes with unusual properties (Stavrakaki and Clahsen, 2009; Varlokosta and Nerantzini, 2013, 2015). By the time they enter elementary school (6 years old) Greek-speaking children have mastered the inflectional paradigms of the language to a large extent, and they can manipulate inflectional morphemes (Rothou and Padeliadu, 2015) while their pragmatic skills make a significant contribution along with vocabulary skills to their narrative performance (Ralli et al., 2021).

Logometro (Mouzaki et al., 2017) was designed to systematically examine young children’s language competence in order to (a) map individual language development paths as well as difficulties, (b) provide a descriptive profile of children’s oral language and literacy competence, and (c) assist in the identification of children with DLD or children who are at risk for SLD. Logometro consists of 24 tasks measuring the various language domains such as phonological awareness and processing, listening comprehension, vocabulary knowledge (receptive and expressive), narrative speech, morphological awareness, pragmatics, as well as emergent literacy skills (letter sound knowledge and invented writing). Almost all domains are assessed through more than one tasks that combine both receptive and expressive language modalities. For example, the vocabulary knowledge score combines child’s achievement on three different tasks -one receptive (word identification) and two expressive (word naming and word definition). Phonological awareness is assessed with four different tasks on both syllabic and phonemic levels (total eight tasks), in order to provide an accurate profile of children’s developing skills that would be taken into account later for intervention, if needed. In a similar fashion morphological awareness is assessed in both the metalinguistic and epilinguistic levels utilizing word production and judgment tasks. All of the aforementioned tasks are presented in a fixed order that has been especially set in order to keep the child’ s interest high and to minimize fatigue.

Test items within each task were determined during extensive pilot testing that included 237 children from four geographically dispersed provinces (Attica, Thessaly, Macedonia, and Crete) of Greece and were a representative proportion of socioeconomic backgrounds. Task items were arranged in an order of ascending difficulty, and they all adhere to floor and ceiling rules that were also determined during the pilot phase. In each task, two practice items were included, that were followed by proper feedback and were repeated up to three times if needed. The task types and the number of items in each task are shown in the Supplementary material.

Logometro is administered through a specially developed Android application for mobile devices that enables proper voicing of directions, easy capturing of children’s responses *via* touch screens and direct recordings (for oral replies), as well as an automatic application of ceiling rules. Once the test administration has been completed, the professionals (the clinicians or educators) automatically retrieve the child’s detailed assessment report from a parallel web-based application. The report includes a precise estimation of the child’s language competence in terms of strengths and weaknesses in each one of the language subcomponents measured, as well as a total language score.

The purpose of the present study was to describe Logometro, as well as to provide information on the psychometric properties of the test and more specifically the internal consistency and test–retest reliability, as well as the construct, convergent, discriminant and predictive validity.

## Materials and methods

### Participants

In total, 926 children between 4 and 7 years old participated in the study. For the evaluation of the normative data, construct, concurrent and predictive validity, 800 typically developing children (384 boys and 416 girls) were assessed. They were attending Prekindergarten (PΚ; *N* = 180, *M* = 4.68 years, SD = 0.45 years), Kindergarten (K; *N* = 269, *M* = 5.6 years, SD = 0.36 years), and Grade 1 (G1; *N* = 351, *M* = 6.62 years, SD = 0.4 years). Age was represented with six subgroups using 6-month intervals, ranging from 4 years, 0 months, 0 days to 6 years, 11 months, 30 days. Each age group involved approximately 100–104 participants, equally distributed between boys and girls. The normative sample did not include children with a diagnosis of DLD, sensory deficits, or developmental delays.

Furthermore, in order to assess the discriminant validity of Logometro, typically developing children’s language competence on the digital language assessment tool was compared with the corresponding performance of children with DLD and children with SLD. Therefore, two more groups of children participated in the study. The first group was 44 children with DLD [*N*_boys_ = 21 boys (*M* = 5.82 years, SD = 1.12), *N*_girls_ = 23 (*M* = 6.02 years, SD = 1.04)], and another group of 82 students at risk for SLD [*N*_boys_ = 52 (*M* = 6.72 years, SD = 0.33), *N*_girls_ = 30 (*M* = 6.68 years, SD = 0.32)] were matched with two subgroups from the typically developing children for gender and age. All the participants had to have a non-verbal intelligence score above >85 as this was measured with Raven’s Educational Colored Progressive Matrices (Sideridis et al., 2015). Furthermore, children in order to be included in the group of DLD they had to score 1,5 SD below the mean on the standardized Test of Expressive Vocabulary (Vogindroukas et al., 2009), while in order for the SLD to be included in the group they had to score 1,5 SD below the mean on the subscale of decoding of the Reading Skills Assessment Test (Padeliadu et al., 2019).

The present study attempted also to explore the predictive validity of Logometro for early reading skills in Year 1. The longitudinal sample included 104 children from the typically developing sample (51 boys and 53 girls) between 4.6 and 6.2 years old, attending Prekindergarten, Kindergarten and 1st Grade.

All the children were speaking Greek as their native language and were randomly selected from schools in rural (19.4%), semi-urban (11.7%), and urban (68.9%) areas of four geographically dispersed provinces (Attica, Thessaly, Macedonia, and Crete) of Greece including a representative proportion of socioeconomic and ethnic background. Parental educational level was reported by 69.2% male and 50.8% female parents. In the male parents’ group 23.8% had graduated from high school and 45.4% were college graduates, while in the female parents’ group, 24.8% had high school diploma and 26% had a college degree.

### Measures

#### Logometro

Logometro is a digital assessment battery that includes tasks measuring systematically a range of oral language skills such as phonological awareness, narrative skills, vocabulary knowledge, morphological awareness, listening comprehension, pragmatics, as well as emerging literacy skills (relevant website www.logometro.gr).

*Phonological awareness* was measured through eight different tasks, namely *identification of similarities, synthesis, segmentation, and elimination*, four at a phoneme level and four at a syllable level. In the *identification of similarities task* (Cronbach’s *α*_syllables_ = 0.84; *α*_phonemes_ = 0.84) each child examined four images and then listened to the label of a target image as well as to the labels of three other images. The aim was to choose the image that began with the same syllable/phoneme as the target image. In the *synthesis task* (Cronbach’s *α*_syllables_ = 0.89; *α*_phonemes_ = 0.93) each child composed words from a series of syllables/phonemes that were pronounced separately. In the *segmentation task*, (Cronbach’s *α*_syllables_ = 0.95; *α*_phonemes_ = 0.95) each child listened to a word and then was asked to analyze/segment it in syllables/phonemes. Accordingly, in the *deletion task* (Cronbach’s *α*_syllables_ = 0.94; *α*_phonemes_ = 0.92) each child listened to a word and then was asked to repeat it by deleting a chank of it (syllable or phoneme, respectively) in order to produce a new, existing, word.

*Narration* comprised of a *retelling task* and a *free narration task*. *Retelling* was measured with the use of a recorded story which was accompanied by six pictures. Each child was invited to listen to a story and then, only by looking at the pictures, was asked to retell it. The story had a narrative genre, and the theme was compatible with the children’s age and background knowledge. In the *free narration* task, each child looked at an illustrated story accompanied by six pictures which were age relevant and was asked to produce a story based on the pictures. Both narrative tasks were analyzed according to microstructure and macrostructure criteria (for more details see Mouzaki et al., 2020).

*Vocabulary knowledge* was assessed with four different tasks: *receptive vocabulary task, naming task, word definition task and listening comprehension*. In the *receptive vocabulary task* (Cronbach’s *α* = 0.88), each child was presented with four different pictures and was asked to choose the picture that best represented the recorded word heard. In the *naming task* (Cronbach’s *α* = 0.72), each child looked at a picture and then was asked to name it. In the *word definition task* (Cronbach’s *α* = 0.93) each child was asked to give a brief definition of a series of words that were heard. *Listening comprehension* was assessed with two tasks. In the *Listening comprehension -directions task* (Cronbach’s *α* = 0.93), each child listened to a direction and had to respond accordingly by choosing the correct image among four images. In the *Listening comprehension -story task* (Cronbach’s *α* = 0.78) each child was presented with a recorded narrative story accompanied by illustrated pictures and was asked to answer questions relevant to the content of the story.

*Morphological awareness* was assessed through five tasks (Diamanti et al., 2017, 2018) that evaluate the implicit and the explicit knowledge of word structure through derivational and inflectional morphology judgment and production. The design and content of the tasks were targeted specifically for the age groups under study, aiming to avoid floor and ceiling effects in assessing the various morphological domains. In the *derivational morphemes production* task (Cronbach’s *α* = 0.94) each child was asked to manipulate derivational morphemes within target words in order to complete sentences correctly according to pictures shown at the same time (e.g., “The sea deepens. The sea is…” requiring “deep”). In the two *inflectional morphemes production tasks* (Cronbach’s *α* = 0.95 and *α* = 0.88, respectively), each child was asked to manipulate the grammatical part of the target item (verb or noun), that was a pseudoword, in order to correspond to a pair of illustrated pictures [e.g., First sentence/picture: “The turtle plays with zagon (pseudoword for wagon); second sentence/picture: The turtle plays with…. requiring “zagons”]. Finally, in the two *inflectional morphemes judgment tasks* (*α* = 0.80 and *α* = 0.84, respectively) each child was presented with a picture displaying either one or two turtles performing an action and had to choose the correct sentence. For example, given a picture of a turtle drinking juice, the two sentences were “the turtle frims (3rd singular) juice” and “the turtle frim (3rd plural) juice.” These two tasks evaluated the correct use of inflectional morphemes (suffixes) at verbs (in present tense, third person, singular and plural) as well as nouns (neutral gender, singular and plural).

Logometro also includes tasks that evaluate children’s emerging literacy skills namely *letter sound knowledge* and *invented writing*. In the *letter sound knowledge task*, the electronic device displayed individual letters (uppercase and small) in a random order and each child was asked to name the sound (voice) they represented. If the child provided as a response the name of the letter, the examiner pointed out that this is the name of the letter and asked the child to name the sound corresponding to that letter. For the *invented writing task*, each child was asked first to write his/her name and to produce a simple sentence (mom I love you). In this task, each child was asked to represent his/her familiarity with letters as well as his/her discoveries of print intricacies. *Invented writing* was evaluated for characteristics that range from print directionality and use of pre-communicative symbols to use of letter-sound correspondences and conventional spelling providing valuable information for the child’s emergent literacy skills. Qualitative analysis was carried out for the emerging literacy tasks. Qualitative ratings of children’s emergent literacy skills based on these tasks are documented along the norm-referenced oral language scores in order to assist with instructional planning.

*Pragmatics* was assessed on the basis of four indicators: (a) the *interpretation of the communicative situation* presented in the picture (Cronbach’s *α* = 0.81), (b) the *intention/ability to communicate* (Cronbach’s *α* = 0.81), (c) the *response to communication* (Cronbach’s *α* = 0.81) and (d) the *interactional skills that are related to the contextual variation* (Cronbach’s *α* = 0.81). Each child was presented with 11 illustrated scenarios corresponding to 21 recorded questions. Each scenario included 1–3 questions that required from each child a verbal response, which was automatically recorded (see Vassiliu et al., 2019, for detailed information).

#### Measures used for the assessment of convergent validity

In order to assess the convergent validity of Logometro, a series of standardized tests were used which are the following: (a) *the* brief Greek version *Receptive Vocabulary Test for Greek Elementary Students*, (b) *the Test of Expressive Vocabulary*, and (c) *the Vocabulary subtest of the WPPSI-III*.

The brief Greek version of *Receptive Vocabulary Test for Greek Elementary students* (Simos et al., 2011), which was adapted from the Peabody Picture Vocabulary Test-Revised (PPVT-R), Form L (Dunn and Dunn, 1981) was used in order to assess each child’s receptive vocabulary skills. Based on pilot data, extensive modifications were made in the structure of the original test to accommodate linguistic and cultural differences (Simos et al., 2011). As in the original task, each child was asked to identify one picture out of four that best represented the word spoken by the examiner. The task was discontinued when the child gave eight incorrect answers within 10 consecutive questions. Short and full forms were closely equivalent as indicated by correlation coefficients ranging between 0.96 and 0.97 across grades.

The standardized *Test of Expressive Vocabulary* (Vogindroukas et al., 2009, the Greek standardization of Renfrew) was used to assess each child’s expressive vocabulary. The test consists of 50 black-and-white images representing familiar objects (Cronbach’s *α* = 0.92). Each child was asked to name each picture shown. The items were presented in ascending difficulty and testing stopped when the child failed to respond correctly in five consecutive items.

The *Vocabulary subtest of the WPPSI-III* (Wechsler, 2002; Sideridis and Antoniou, 2015) was used in order to assess the child’s word knowledge and verbal concept formation. Each child was asked to give verbal definitions for 20 words that the examiner read aloud and to name five picture items (Cronbach’s *α* = 0.84). For picture items, each child was asked to name pictures that were displayed by the examiner. The task was discontinued when the child gave six incorrect consecutive responses.

#### Measures used for the assessment of discriminant and predictive validity

In order to assess the discriminant validity of Logometro, typically developing children’s language competence on Logometro was compared with the corresponding performance of children with DLD and children with SLD. As described above, a series of standardized tests were used, which are the following: (a) *Raven’s Educational Colored Progressive Matrices*, (b) *the Test of Expressive Vocabulary* and (c) *the Reading Skills Assessment Test*.

*Non-verbal intelligence* was evaluated through the Greek standardization of the *Raven’s Educational Colored Progressive Matrices (Raven-CPM) test* (Sideridis et al., 2013), which includes 36 problems, divided into three sets of increasing difficulty (Cronbach’s *α* = 0.82). Raven-CPM problems consist of either a geometric shape that lacks a segment or a series of four shapes, one of which is missing. Each child was asked to choose the correct one from the six alternative shapes.

The *Reading Skills Assessment Test* (*RSAT*, Padeliadu et al., 2019) was used in order to assess child’s reading competence. The *word decoding* subscale consists of 57 words (Cronbach’s *α* = 0.95), with an increasing number of syllables (2–7) and semantic complexity. The child was asked to read aloud the words presented. The task was discontinued when the child read incorrectly five consecutive words. The *pseudoword decoding* subscale consists of 40 pseudowords (Cronbach’s *α* = 0.91), with an increasing number of syllables (2–6) and phonological complexity. The child was asked to read aloud the pseudowords presented. The task was discontinued when the child read incorrectly five consecutive pseudowords. The *fluency* task consisted of a 247-word narrative text. The child was asked to read the narration quickly and accurately within 1 min. The *reading comprehension* task consisted of three narrative and expository texts, which were presented in an ascending semantic difficulty. The child was asked to answer in total 21 questions related to the literal and explicit meaning of the texts (Cronbach’s *α* = 0.89).

Spelling ability was assessed using the standardized spelling-to-dictation *Word Spelling Test for Greek Elementary School Students* (Mouzaki et al., 2007). The test included 60 words dictated in isolation and in a sentence and it is discontinued when the child gives six consecutive misspelled responses (Cronbach’s *α* = 0.945).

### Procedures

The study protocol was approved by the Greek Ministry of Education and the Research Ethics Committee of the University of Crete. Children participating in the study had written consent from their parents/guardians. The assessment took place individually in a quiet room at school by a specially trained postgraduate psychology or special education student after obtaining the written parental consent and child’s oral assent. The examinee was allowed to rest when tired by giving breaks. Testing time ranged between two to three 40-min sessions within 2 weeks. The predictive validity was estimated by individual assessments completed 3 times within an 18-month-period (mid-Kindergarten, beginning and end of 1st grade).

All answers were recorded *via* pencil and paper (for the IQ, vocabulary, reading and spelling tests) or on the tablet (Logometro®). Task administration was accomplished through the specially developed Android application for tablets. The application had a familiar and attractive interface in order to attract the child’s attention and eagerness to complete the assessment. Except detailed directions, each task incorporated practice examples, and suitable feedback for the child. Most of the tasks were automatically discontinued when the child provided a specific number of consecutively incorrect answers. Tablets enabled the child to record the answer directly by touching the screen or by recording her/his oral responses. Task advancement was adjusted to child’s pace by the examiner who also recorded the correctness of the response by selecting certain symbols (without being noticed by the child) and forwarded the process. The tasks in need of manual scoring according to preset criteria were scored later by the authors in the parallel web-based application.

### Data analyses

Omega reliability was engaged as an estimate for internal consistency reliability for each latent construct. Bivariate correlations were utilized to evaluate test–retest reliability and convergent validity, while t-tests were run to evaluate discriminant validity. Hierarchical multiple regression analysis was run to determine whether participants’ oral language performance in the middle and end of kindergarten year was predictive of reading and spelling at the end of 1st grade. Factorial validity was evaluated using Confirmatory Factor Analysis (CFA) with measured language skills combined to assess latent language subconstructs. Given the mixture of item formats, the Weighted Least Squares with Mean and Variance (WLSMV) adjusted estimator was utilized which is appropriate for modeling discrepancies of measured components from normality at both the univariate and multivariate level. Omnibus model fit was evaluated using (a) the chi-square test, (b), unstandardized residuals (i.e., Root Mean Square Error of Approximation-RMSEA-Steiger and Lind, 1980; Steiger, 1990), and (c) descriptive fit indices (i.e., the Comparative Fit Index and the Tucker-Lewis Index). For the chi-square statistic, support of the null hypothesis would be indicative of perfect model fit and an errorless model. Across most instances, however, the chi-square test is extremely sensitive to monitor discrepancies between estimated and actual model fit, thus, it is given little precedence. Most useful are the unstandardized residuals for which estimates below 5% are indicative of “exact fit” based on the thesis of MacCallum et al. (1996). Acceptable values are considered those that are less than 8%. Last, conventions for most descriptive fit indices point to acceptable model fit when values are greater than 0.900 or 0.950 or when values of residuals and fit indices agree in favor of a single conclusion (Hu and Bentler, 1995, 1999). All analyses were run using Mplus 8.7 and the level of significance was set to 5% for a two-tailed test.

## Results

### Construct validity

**Figure 1:**
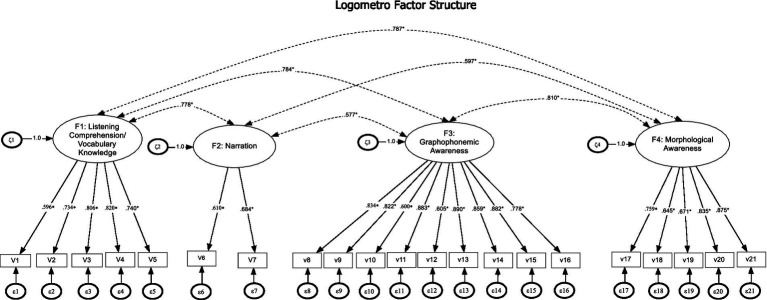
Final model for the measurement of oral language skills (Logometro) reflecting four intercorrelated latent constructs.

Initially, the instrument was constructed to assess seven domains, namely, *phonological awareness, narration, vocabulary knowledge, listening comprehension, morphological awareness, emergent literacy (letter-sound knowledge, invented writing) and pragmatics*. However, the pragmatics task, as well as the invented writing tasks, due to being single measured variables, were not included in the latent variable modeling used. Furthermore, the latent correlation between Listening Comprehension (LC) and Vocabulary Knowledge (VK) was 1.0 and, similarly, the correlation between Phonological Awareness (PA) and Letter-sound Knowledge (LK) was 0.986. To further conclude collapsing highly correlated constructs, the original conceptualization was contrasted to a model where the two highly correlated factors were constrained to be correlated at unity. Non-significance between the two nested models would be indicative of the “null” hypothesis that the correlation between the two constructs can be equal to 1. Results after constraining the relationship between LC and VK to be 1 indicated a non-significant difference chi-square test [Diff*χ*^2^(1) = 2. 769, *p* = n.s.] and the same was true after specifying a perfect correlation between phonology and letter sound knowledge [Diff*χ*^2^(1) = 1.841, *p* = n.s.]. Thus, Listening Comprehension and Vocabulary Knowledge were combined to comprise the first latent factor and Phonological Awareness and Letter-sound Knowledge the 3d factor termed Graphophonemic Awareness, as shown in Figure 1.

**Table 1:** Model comparison in search of the optimal structure of Logometro.

Model tested	Chi-square	D.F.	RMSEA	CFI	TLI	Model comparison	ΔChi-square	Δ-DF	Δ-CFI	Δ-TLI
M1: Unidimensional model	2692.038^***^	190	0.150	0.797	0.776	–	–	–	–	–
M2: Hierarchical model	629.231^***^	185	0.064	0.964	0.959	M2 vs. M1	2062.807	5	0.167	0.183
M3: Four-factor correlated	567.078^***^	183	0.058	0.972	0.968	M3 vs. M2	62.153	2	0.08	0.09
M4: Bifactor model	472553^***^	168	0.056	0.957	0.947	N/A	N/A	N/A	N/A	N/A

Delta CFI and TLI values are in absolute terms. N/A, not applicable because the bifactor model is not nested within the four-factor correlated model.

Consequently, a four-construct representation as shown in Table 1 provided the most parsimonious solution with the present data. All measurement paths were significant, unstandardized residual values were acceptable (between 5% and 8%) and descriptive fit index values were over 0.950. As a final test of validity, this optimal structure (M3) was contrasted to a unidimensional framework (M1), a hierarchical model (M2) and a bifactor model. Results indicated that a unified construct was the least appropriate representation for the measurement of Logometro skills as it was associated with 2,124 points of misfit (in chi-square terms) compared to the four-factor correlated model. Furthermore, the hierarchical model was also suboptimal as it was associated with significantly higher misfit using both global statistics (chi-square) and descriptive fit indices as well. Thus, the lower order factor correlations were not fully accounted for by the higher order factor. As a last check, a bifactor model was utilized positing a general factor and four specific factors. The bifactor model was not nested with the four-factor correlated model thus direct comparisons were not possible. However, besides having relatively lower in magnitude CFI/TLI values, there were two interpretation problems with the bifactor model. First, although the general factor was supported, no specific factor was fully supported with either having some specific factors extinct and others having some relevant items. Second, some item slopes in the specific factors became negative in the bifactor model, which represents an important indication of model misfit and a serious cause for concern and item removal (Dunn and McCray, 2020). Thus, it was concluded that a four-factor correlated structure (Listening comprehension/Vocabulary Knowledge, Narration, Graphophonemic Awareness, Morphological Awareness) best describes the latent oral language skills of 4–7 years old children. Interestingly, the model did not include any residual correlations or other alterations (additional estimates) so that not to capitalize on chance.

**Figure 2:**
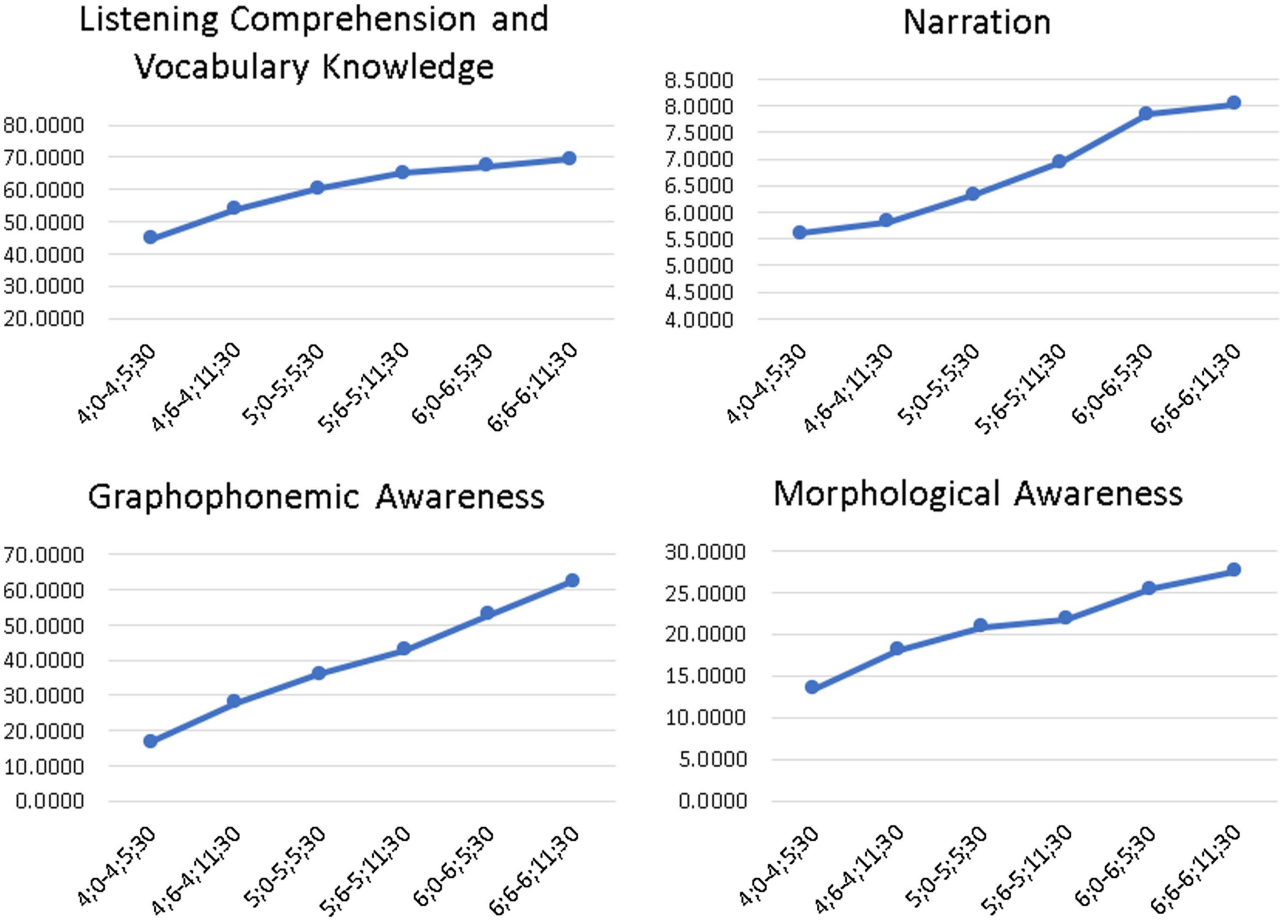
Developmental trajectories of the four domains of the Logometro for the different age groups.

As a final test of the monotonicity hypothesis that as age increases so does achievement, Figure 2 displays the developmental trajectories of the four domains of the logometro for the different age groups.

### Convergent validity

Bivariate correlations were estimated between (a) the receptive vocabulary task from Logometro against the brief Greek version of PPVT (Simos et al., 2011), (b) the naming task from Logometro versus the standardized test of expressive vocabulary (Vogindroukas et al., 2009), and (c) the definition task from Logometro versus the same construct from the Greek standardization of WPPSI-III (Sideridis and Antoniou, 2015). Using Pearson’s *r*, results indicated significant positive correlations between all the different pairs of measurements (Table 2). Furthermore, value of *p* were supplemented by effect size indicators as suggested by Cohen (1992) with *r*-values of 0.1, 0.3, and 0.5 reflecting small, medium, and large effects, respectively.

**Table 2:** Correlations between Logometro and relevant constructs as evidence of convergent validity.

Model tested	*r*	C.I._95%_	*p*	Effect size
Vocabulary constructs	0.761	0.665–0.833	<0.001	Large
Naming constructs	0.807	0.726–0.866	<0.001	Large
Definition constructs	0.794	0.708–0.857	<0.001	Large
Word recognition	0.761	0.665–0.833	<0.001	Large

### Discriminant validity

In order to investigate discriminant validity, mean differences were estimated between Logometro constructs in typically developing children versus children at risk for SLD as well as between typically developing children and children with DLD. Using summed raw scores, results pointed to the better performance of the typically developing children over those at risk for SLD on the first factor (Vocabulary Knowledge and Listening Comprehension) [*t*(162) = 1.997, *p* = 0.047], the second-factor, Narration [*t*(162) = 4.459, *p* < 0.001], but also the third factor Graphophonemic Awareness [*t*(162) = 3.225, *p* = 0.002]. There were no significant differences on Morphological Awareness between the two groups. When contrasting typically developing children with those with DLD again the first group of children had a statistically significantly better performance on Vocabulary Knowledge and Listening Comprehension [*t*(86) = 4.226, *p* < 0.001], on Narration [*t*(86) = 2.220, *p* = 0.029], on Graphophonemic Awareness [*t*(86) = 5.556, *p* < 0.001] and on Morphological Awareness [*t*(86) = 5.092, *p* < 0.001] than the children with DLD.

### Predictive validity

To determine whether participants’ performance on Logometro in the middle and end of kindergarten year predicted their performance on word and pseudoword accuracy, reading fluency, reading comprehension, and spelling at the end of the 1st Grade, a hierarchical multiple regression analysis was carried out. There were high concurrent inter correlations among the composite scores. Emergent literacy skills (as measured with the letter-sound task and invented writing) correlated significantly with phonological awareness during both measurement times (Tables 3–5). Composite scores for emergent literacy skills, morphological and phonological skills made a further significant contribution to the prediction of reading outcomes (word and pseudoword accuracy, reading fluency, reading comprehension, and spelling) in Step 2 of the model, after controlling for all the other variables entered together in Step 1 (Tables 6, 7).

**Table 3:** Inter-correlations among composite scores (Time 1 and Time 2).

Composites	Receptive language	Expressive language	Morphological awareness	Phonological awareness	Literacy skills
Receptive language	–	0.504	0.398	0.237	0.148
Expressive language	0.402	–	0.457	0.345	0.290
Morphological awareness	0.285	0.421	–	0.551	0.394
Graphophonemic awareness	0.338	0.372	0.496	–	0.725
Literacy skills	0.382	0.308	0.451	0.618	–

**Table 4:** Correlations between composite scores (Time 1) and later reading and spelling.

Composites	Receptive language	Expressive language	Morphological awareness	Phonological awareness	Literacy skills
Reading accuracy	0.097	0.251	0.467	0.292	0.482
Decoding words	0.112	0.218	0.488	0.344	0.411
Reading fluency	0.110	0.152	0.174	0.373	0.397
Reading comprehension	0.307	0.346	0.444	0.319	0.392
Spelling	0.140	0.127	0.304	0.422	0.410

**Table 5:** Correlations between composite scores (Time 2) and later reading and spelling.

Composites	Receptive language	Expressive language	Morphological awareness	Phonological awareness	Literacy skills
Reading accuracy	0.205	0.266	0.353	0.367	0.393
Decoding words	0.191	0.233	0.302	0.374	0.317
Reading fluency	0.252	0.200	0.211	0.379	0.311
Reading comprehension	0.417	0.361	0.314	0.364	0.342
Spelling	0.278	0.270	0.228	0.381	0.230

**Table 6:** Hierarchical multiple regression analysis results (Time 1: middle of kindergarten year).

Time 1	*R*^2^ change (p)				
	Early literacy skills	Expressive language	Receptive language	Morphological awareness	Phonological awareness
Reading words	**0.110 (0.00)**	0.005 (0.38)	0.017 (0.11)	**0.073 (0.00)**	0.008 (0.27)
Pseudowords	**0.038 (0.02)**	0.000 (0.85)	0.007 (0.33)	**0.090 (0.00)**	0.001(0.76)
Fluency	**0.052 (0.01)**	0.001 (0.75)	0.004 (0.48)	0.003 (0.52)	0.030 (0.06)
Comprehension	0.023 (0.08)	0.013 (0.19)	0.008 (0.31)	**0.052 (0.01)**	0.001 (0.77)
Spelling	**0.032 (0.04)**	0.005 (0.43)	0.002 (0.58)	0.010 (0.27)	**0.034 (0.04)**

Values in bold letters show significant correlations.

**Table 7:** Hierarchical multiple regression analysis results (Time 2: beginning of 1st grade).

Time 1	*R*^2^ change (prob.)				
	Early literacy skills	Expressive language	Receptive language	Morphological awareness	Phonological awareness
Reading words	**0.031 (0.05)**	0.003 (0.52)	0.003 (0.53)	0.017 (0.14)	0.001 (0.74)
Pseudowords	**0.038 (0.02)**	0.002 (0.59)	0.005 (0.45)	0.005 (0.44)	0.020 (0.13)
Fluency	0.003 (0.53)	0.000 (0.96)	0.025 (0.09)	0.003 (0.55)	**0.037 (0.03)**
Comprehension	0.013 (0.19)	0.009 (0.28)	**0.063 (0.00)**	0.000 (0.96)	0.006 (0.35)
Spelling	0.004 (0.48)	0.007 (0.37)	0.017 (0.14)	0.004 (0.49)	**0.068 (0.00)**

Values in bold letters show significant correlations.

### Reliability

Test–retest reliability of Logometro was assessed during two consecutive measurements in a 2-week interval. Pearson correlation analysis was conducted on the test and retest scores of 33 typically developing children, and the results showed that test–retest reliability of each Logometro subscale was between 0.734 and 0.964. More specifically, statistically high significant correlations between the two times measurements were found for the Vocabulary Knowledge and Listening Comprehension tasks (*r*_test-retest_ = 0.923, *p* < 0.001), as well as for the Graphophonemic Awareness (*r*_test-retest_ = 0.964, *p* < 0.001) and Morphological Awareness (*r*_test-retest_ = 0.950, *p* < 0.001), while statistically significant medium correlations emerged for the Narration task (*r*_test-retest_ = 0.734, *p* < 0.001).

Using the omega coefficient which is most appropriate for non-tau-equivalent measures, results indicated acceptable internal consistency reliability for all four constructs of the Logometro. Table 8 presents the omega estimates for (a) *Listening Comprehension—Vocabulary Knowledge*, (b) *Narration*, (c) *Graphophonemic Awareness*, and (d) *Morphological Awareness*.

**Table 8:** Internal consistency reliability using McDonald’s omega coefficient.

Logometro constructs	Omega
Listening comprehension—Vocabulary knowledge	0.865
Narration	0.625
Graphophonemic awareness	0.895
Morphological awareness	0.868

## Discussion

The aim of the current study was to describe Logometro®—a digital language assessment battery, for Greek-speaking children, aged 4–7 years- as well as its psychometric properties. Logometro aims at measuring young children’s language skills with the goal of providing a valid screening tool for their language strengths and weaknesses. It consists of 24 tasks measuring various oral language domains such as phonological awareness and processing, oral language comprehension, vocabulary knowledge (receptive and expressive), narrative skills, morphological awareness, and pragmatics as well as emergent literacy skills.

Several important findings emerged. First, looking at the factorial validity it was found that the originally thought seven-factor structure was best described by four latent dimensions due to the high correlation between Listening Comprehension and Vocabulary Knowledge and between Phonology and Letter-sound Knowledge. The distinction between Listening Comprehension and Vocabulary Knowledge tasks was not supported by the tests of factorial validity resulting to a merged factor. Such finding confirms prior conceptualization of oral language domains for this age group that does not distinguish oral language (Listening Comprehension) from lexical/semantic knowledge (Vocabulary Knowledge) and supports the consolidation of foundational skills involving words, sentences, and discourse skills in preschoolers and early years children (Mouzaki et al., 2020). Furthermore, this finding is partially justified by the high similarity among the respective tasks: both listening comprehension and receptive vocabulary tasks invited children to look at four different pictures and then to choose the picture that best represented the word -or the sentence- that was heard. On the other hand, the expressive vocabulary task of word definitions is much broader than an estimate of the extend of semantic representations and categorizations among them, posing high cognitive demands, in order to form an appropriate definition for any given word. Such demands are comparable to requirements for comprehending short stories presented orally (and answering explicit and implicit questions) as directed in the listening comprehension task- story. The strong association between phonological awareness skills and letter-sound knowledge that led to the merged factor *Graphophonemic Awareness* is in complete agreement with previous evidence involving both English (Burgess and Lonigan, 1998; Lonigan et al., 2000; Foy and Mann, 2006) and Greek-speaking children of the same age (Manolitsis and Tafa, 2011) supporting their bidirectional relation. Thus, aggregating those terms resulted in two fewer latent variables with more precise assessments (with less measurement error) and a more parsimonious assessment of language skills in the early years. Model fit of the final model was excellent with residual values approximating “exact fit” as per MacCallum et al. (1996) conceptualization.

Furthermore, evidence on convergent validity indicated high correlations between the Logometro sub-scales and respective constructs from validated and normed instruments in the Greek population. Interestingly, these estimates were not extremely high so that they would indicate overlap at the level of duplicating another measure with no additional information.

Additional evidence on discriminant validity indicated that known groups for which different language ability was to be expected were clearly differentiated using Logometro. In more detail, typically developing children significantly outperformed children with DLD in all language domains consistent with previous research evidence (Bishop et al., 2016, 2017). Similarly, children at risk for SLD scored significantly lower than their typical peers on all factors except from Morphological Awareness. Even though this result contrasts from some earlier studies there are few possible explanations for the absence of differences between the two groups. Previous evidence from shallow orthographies has also shown that Italian-speaking children with specific reading disability did not differ from their age-matched controls in morphological awareness tasks as both groups reached ceiling performance in a task involving plural generation of nouns and determiners (Vender et al., 2017). In a recent study involving Greek-speaking children with and without literacy difficulties, it was shown that the differences in morphological awareness growth that was found between the two groups, could be accounted for in part by the different measures used (word analogies, compound word production, etc.) and the older age of participating children (Kargiotidis et al., 2021). Similarly, other studies pointing to morphological awareness differences between typical and Learning-Disabled children groups have involved older children or participants who were mainly impaired in reading comprehension skills (Rothou and Padeliadu, 2019; Kargiotidis et al., 2021; Rothou, 2021) providing a mixed pattern of results and underlining the need for further research.

Predictive validity findings are consistent with previous evidence verifying well known predictors of literacy achievement (Kendeou et al., 2009; Torppa et al., 2010; Melby-Lervåg et al., 2012; Catts et al., 2016; Pittas, 2018). In more detail, phonological awareness accounted for variance in spelling skill and it also explained the variance in reading outcomes as supported by previous studies in transparent orthographies (Plaza and Cohen, 2007; Landerl et al., 2019). The non-significant contribution of phonological awareness on reading skill is probably due to the fact that at this age phonological awareness variance is largely shared with morphological awareness variance which ended up capturing most of the contribution to reading outcomes as an overall metalinguistic skill (Diamanti et al., 2017). Preschool morphological awareness predicted early reading accuracy beyond phonological awareness and vocabulary skills, suggesting the significant contribution that early understanding of word structure has upon reading development (Diamanti et al., 2017). Morphological skills also found to play a significant role in reading comprehension consistently with similar investigations in other transparent orthographies (Manolitsis et al., 2017; de Freitas et al., 2018; Desrochers et al., 2018). However, previous findings do not describe unique predictive relation to reading during the transition to first grade (i.e., Storch and Whitehurst, 2002). The link between vocabulary knowledge and reading comprehension found could be explained (a) by previous findings showing that oral vocabulary is a strong longitudinal predictor of reading comprehension especially in middle and upper elementary grades (Protopapas et al., 2007; Kim et al., 2014; Suggate et al., 2014) and (b) by the transparency of the orthographic system as children learning to read Greek in a more effortless and timely manner than learners of the English orthography and they are exposed faster to more complex reading texts (Padeliadu and Antoniou, 2014). Finally, letter sound knowledge is one of the best predictors of children’s early reading proficiency (Hulme et al., 2012; Clayton et al., 2020) and it appears to have an indirect association with reading acquisition through phonological sensitivity. There is some evidence that letter knowledge and phonological sensitivity may be reciprocally related (Burgess and Lonigan, 1998; Lonigan et al., 2000; Foy and Mann, 2006) as well as there is evidence that invented spelling is a good vehicle for practicing phonological sensitivity and knowledge of letter-sound correspondences both of which are directly related to decoding.

The previously mentioned results along with the reliability results, have further strengthened our confidence that digital assessments hold several advantages (e.g., reliability and economy of administration, interest, portability and ease, etc.), which have been frequently highlighted in the relevant literature (Frank et al., 2016; Neumann and Neumann, 2019). The reliability results highlighted the stability of the measurement with the Logometro over time and the internal consistency of its components. Thus, collectively, Logometro possesses the requisite psychometric properties to be used for the valid assessment of language skills in the early years. The use of computerized language assessment tools such as Logometro guarantees the gathering of accurate responses (in time and accuracy), which are crucial indicators for the screening of language strengths and weaknesses (Richter et al., 2013). Their highest possible usage is in cases where the available qualified staff is not sufficient to meet the needs of the general school population. Furthermore, digital psychometric tools, such as Logometro, are easy to administer, provide results that are easy to score, as well as produce automatically digital reports. Mobile devices, such as tablets, are popular with children and easy to manipulate thus, they increase children’s motivation to participate and complete the tasks (Dujardin et al., 2021).

Mobile devices are also easy and appealing for non-typically developing children such as children with DLD, with reading difficulties, or with Learning Disabilities in the case of children at the beginning of Grade 1 (Dujardin et al., 2021). An important finding of the current study was that Logometro provides not only the child’s language profile, but also discriminative validity for distinguishing typical development from SLD and DLD status. As Bishop and Snowling (2004) highlighted there is a relationship between neurodevelopmental disorders of language and reading and, there are two dimensions of variation. Phonological skills and oral language competence are the main dimensions of differentiation. SLD are related to poor phonological skills and subsequently poor decoding whereas DLD is linked to poor reading comprehension. Nevertheless, both neurodevelopmental disorders can co-occur even though DLD can coincide with poor phonology or competent phonological awareness skills (Nation et al., 2004). Thu, Logometro® enables clinicians and/or educators to examine in a complete and thorough manner the child’s language development profile and decide upon provision of referrals or educational interventions.

Computer-based screening for oral language difficulties is feasible, practical -in comparison to paper and pencil tests- and psychometrically suitable for younger children. Early identification of language difficulties helps to provide children with suitable interventions. It is well known that if a child’s language difficulties are encountered before the beginning of formal reading instruction, it is possible for the child to overcome the risk of a positive diagnosis (Catts et al., 2005). Nevertheless, parents, clinicians, educators, and administrators, should be aware that a computer-based assessment of language cannot provide a diagnosis on its own. Language assessment reports attained by Logometro, or similar digital screening tools can only be a first step toward identifying risk-status and seeking further assessments in order to guide an appropriate intervention plan.

## Data availability statement

The raw data supporting the conclusions of this article will be made available by the authors, without undue reservation.

## Ethics statement

The studies involving human participants were reviewed and approved by the Greek Ministry of Education. Written informed consent to participate in this study was provided by the participants’ legal guardian/next of kin.

## Author contributions

FA, AR, AM, VD, and SP contributed to the design and development of the study and provided access to the study population. FA organized the database. FA and AM performed the statistical analysis. FA, AR, and AM participated in the interpretation of the results, as well as in the choice of theory. All authors contributed to the article and approved the submitted version.

## Funding

The pilot phase of this research was supported in part by a post-doctoral research fellowship to VD in the context of research program “The Foundation of Reading and Writing in a Transparent Orthography: Oral Language Development and Early Literacy Skills” funded by the University of Crete Special Account of Research (PI: AM).

## Conflict of interest

The authors declare that the research was conducted in the absence of any commercial or financial relationships that could be construed as a potential conflict of interest. The preschool measures reported here form part of a commercially available screening battery (Logometro, produced by InteLearn Multimedia Educational Applications) designed by the authors, who receive part of the proceeds from its use.

## Publisher’s note

All claims expressed in this article are solely those of the authors and do not necessarily represent those of their affiliated organizations, or those of the publisher, the editors and the reviewers. Any product that may be evaluated in this article, or claim that may be made by its manufacturer, is not guaranteed or endorsed by the publisher.

## Supplementary materials

The Supplementary materials for this article can be found online at: https://www.frontiersin.org/articles/10.3389/fpsyg.2022.900600/full#supplementary-material
