# Supplementary material for: Logometro®: The psychometric properties of a norm-referenced digital battery for language assessment of Greek-speaking 4–7 years old children
Source: Front Psychol. 2022 Jul 26;13:900600. doi: 10.3389/fpsyg.2022.900600 (PMC9361844; doi:10.3389/fpsyg.2022.900600)
Supplement: Supplementary file 1 [file Table_1.DOCX]

| **Language Domain** | **Assessment level** | **Task** | **N**  **Items** |
| --- | --- | --- | --- |
| Phonological Awareness | Syllabic | Identification of similarities | 7 |
|  |  | Synthesis | 5 |
|  |  | Segmentation | 6 |
|  |  | Elimination | 7 |
|  | Phonemic | Identification of similarities | 7 |
|  |  | Synthesis | 7 |
|  |  | Segmentation | 6 |
|  |  | Elimination | 7 |
| Narrative Skills |  | Free Narration | 6 |
|  |  | Retelling | 6 |
| Vocabulary Knowledge |  | Receptive vocabulary | 30 |
|  |  | Naming | 20 |
|  |  | Word definition | 28 |
| Listening Comprehension |  | Directions | 16 |
|  |  | Story questions | 6 |
| Morphological Awareness | Derivational | Morphemes - production | 9 |
|  | Inﬂectional | Morphemes - production - verbs | 8 |
|  |  | Morphemes - production - nouns | 8 |
|  |  | Morphemes - judgment - verbs | 8 |
|  |  | Morphemes - judgment - nouns | 3 |
| Pragmatics |  | 4 indicators in one task: Interpretation of the communicative situation  Intention/ability to communicate  Response to communication  Interactional skills related to the contextual variation | 22 |
| Early Literacy |  | Letter-sound knowledge | 23 |
|  |  | Invented writing – child’s name | 1 |
|  |  | Invented writing – simple sentence | 1 |
